# (*E*)-3-(Anthracen-9-yl)-1-(4-bromo­phen­yl)prop-2-en-1-one^1^

**DOI:** 10.1107/S1600536809003122

**Published:** 2009-01-31

**Authors:** Thitipone Suwunwong, Suchada Chantrapromma, Chatchanok Karalai, Paradorn Pakdeevanich, Hoong-Kun Fun

**Affiliations:** a Department of Chemistry and Center of Excellence for Innovation in Chemistry, Faculty of Science, Prince of Songkla University, Hat-Yai, Songkhla 90112, Thailand; b Crystal Materials Research Unit, Department of Chemistry, Faculty of Science, Prince of Songkla University, Hat-Yai, Songkhla 90112, Thailand; c Department of Physics, Faculty of Science, Prince of Songkla University, Hat-Yai, Songkhla 90112, Thailand; d X-ray Crystallography Unit, School of Physics, Universiti Sains Malaysia, 11800 USM, Penang, Malaysia

## Abstract

In the title mol­ecule, C_23_H_15_BrO, the prop-2-en-1-one unit is planar and it makes dihedral angles of 20.9 (1) and 45.8 (1)°, respectively, with the 4-bromo­phenyl ring and the anthracene ring system. The interplanar angle between the 4-bromophenyl ring and the anthracene ring system is 35.52 (7)°. In the crystal structure, mol­ecules are linked into dimers by C—H⋯Br hydrogen bonds, and the dimers are linked into a zigzag network parallel to the *bc* plane by weak C—H⋯O hydrogen bonds and C—H⋯π inter­actions involving the central benzene ring of the anthracene ring system.

## Related literature

For bond-length data, see: Allen *et al.* (1987 ▶). For related structures, see: Ng *et al.* (2006 ▶); Patil *et al.* (2006 ▶); Patil, Chantrapromma *et al.* (2007 ▶); Suwunwong *et al.* (2009 ▶). For background and applications of chalcones, see: Jung *et al.* (2008 ▶); Patil, Chantrapromma *et al.* (2007 ▶); Patil, Dharmaprakash *et al.* (2007 ▶); Patil & Dharmaprakash (2008 ▶); Prasad *et al.* (2008 ▶).
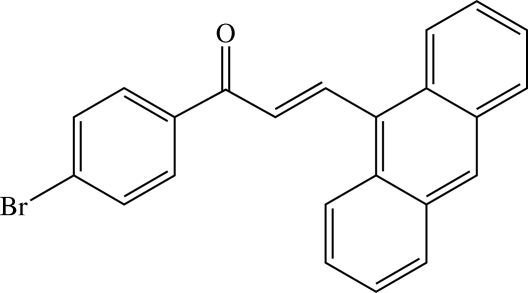


## Experimental

### 

#### Crystal data

C_23_H_15_BrO
*M* *_r_* = 387.25
Monoclinic, 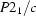

*a* = 5.3792 (1) Å
*b* = 19.1030 (4) Å
*c* = 16.3005 (4) Å
β = 95.944 (1)°
*V* = 1666.02 (6) Å^3^
*Z* = 4
Mo *K*α radiation
μ = 2.47 mm^−1^
*T* = 100.0 (1) K
0.57 × 0.27 × 0.15 mm

#### Data collection

Bruker SMART APEXII CCD area-detector diffractometer
Absorption correction: multi-scan (*SADABS*; Bruker, 2005 ▶) *T*_min_ = 0.331, *T*_max_ = 0.714
29994 measured reflections
4866 independent reflections
3803 reflections with *I* > 2σ(*I*)
*R*_int_ = 0.036

#### Refinement

*R*[*F* ^2^ > 2σ(*F* ^2^)] = 0.032
*wR*(*F* ^2^) = 0.076
*S* = 1.02
4866 reflections
226 parameters
H-atom parameters constrained
Δρ_max_ = 0.53 e Å^−3^
Δρ_min_ = −0.54 e Å^−3^

### 

Data collection: *APEX2* (Bruker, 2005 ▶); cell refinement: *APEX2*; data reduction: *SAINT* (Bruker, 2005 ▶); program(s) used to solve structure: *SHELXTL* (Sheldrick, 2008 ▶); program(s) used to refine structure: *SHELXTL*; molecular graphics: *SHELXTL*; software used to prepare material for publication: *SHELXTL* and *PLATON* (Spek, 2003 ▶).

## Supplementary Material

Crystal structure: contains datablocks global, I. DOI: 10.1107/S1600536809003122/ci2756sup1.cif

Structure factors: contains datablocks I. DOI: 10.1107/S1600536809003122/ci2756Isup2.hkl

Additional supplementary materials:  crystallographic information; 3D view; checkCIF report

## Figures and Tables

**Table 1:** Hydrogen-bond geometry (Å, °) *Cg*1 is the centroid of the C18–C23 ring.

*D*—H⋯*A*	*D*—H	H⋯*A*	*D*⋯*A*	*D*—H⋯*A*
C8—H8*A*⋯O1^i^	0.93	2.42	3.308 (2)	159
C13—H13*A*⋯O1^ii^	0.93	2.57	3.288 (2)	135
C21—H21*A*⋯Br1^iii^	0.93	2.93	3.4722 (19)	119
C9—H9*A*⋯*Cg*1^iv^	0.93	2.83	3.4479 (18)	125

Symmetry codes: (i) 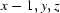; (ii) 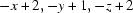; (iii) 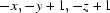; (iv) 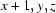.

**Fig. 1.**
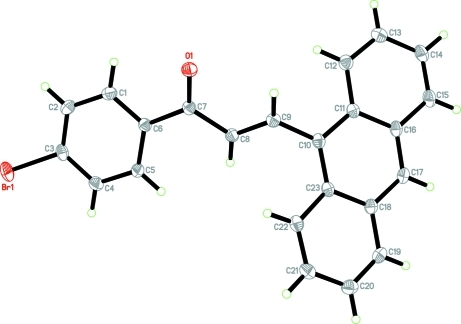
The molecular structure of the title compound, showing 60% probability displacement ellipsoids and the atom-numbering scheme.

**Fig. 2.**
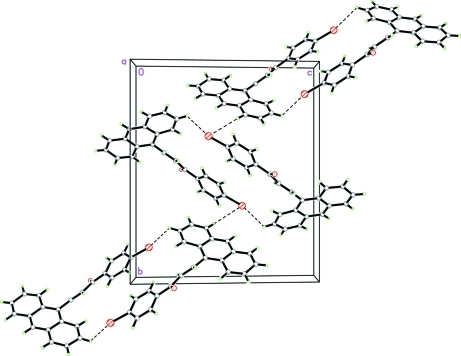
Part of the crystal packing of the title compound, viewed along the a axis, showing hydrogen-bonded (dashed lines) dimers.

## supplementary crystallographic information

### Comment

Chalcones are compounds which have a wide range of applications covering from non-linear optical (Patil & Dharmaprakash, 2008) and electro-active fluorescent materials (Jung *et al.*, 2008) to materials with various biological activities (Prasad *et al.*, 2008). Our previous work (Patil Dharmaprakash *et al.*, 2007) has reported that 1-(4-bromophenyl)-3-(2,4,5-trimethoxyphenyl)-propenone shows efficient second-order nonlinear optical properties. The various interesting properties of chalcone derivatives lead us to synthesize the title chalcone derivative in order to study its photoluminescence and antimicrobial activities.

The molecule of the title chalcone derivative (Fig. 1) exists in an *E* configuration with respect to the C8═C9 double bond [1.333 (2) Å]. The anthracene ring system is planar, with atom C21 deviating a maximum of 0.147 (2) Å. The molecule is twisted as indicated by the interplanar angle between 4-bromophenyl ring and anthracene ring system of 35.52 (7)°, and torsion angles C5–C6–C7–C8 of 22.9 (1)° and C8–C9–C10–C23 of -50.2 (3)°. The pro-2-en-1-one unit (C7-C9/O1) is planar as evidenced by the torsion angle O1–C7–C8–C9 of 0.1 (3)°. The O1/C6-C9 plane makes dihedral angles of 20.9 (1)° and 45.8 (1)°, respectively, with the 4-bromophenyl ring and anthracene ring system. The bond distances show normal values (Allen *et al.*, 1987) and are comparable with those observed in related structures (Ng *et al.*, 2006; Patil *et al.*, 2006; Patil, Chantrapromma *et al.*, 2007; Suwunwong *et al.*, 2009).

In the crystal packing (Fig. 2), the molecules are linked into dimers by weak C—H···Br interactions (Table 1) and the dimers are further linked into a zigzag network parallel to the *bc* plane by weak C—H···O and C—H···π interactions (Table 1).

### Experimental

The title compound was synthesized by the condensation of anthracene-9-carbaldehyde (0.01 mol) with 4-bromoacetophenone (0.01 mol) in ethanol (40 ml) in the presence of NaOH (10 ml, 10%). After stirring for 2 h, a yellow solid appeared and was then collected by filtration, washed with distilled water, dried and purified by repeated recrystallization from acetone. Yellow plate-shaped single crystals of the title compound suitable for *X*-ray structure determination were obtained by slow evaporation of an acetone solution at room temperature after several days.

### Refinement

All H atoms were placed in calculated positions, with C-H = 0.93 Å, *U*_iso_ = 1.2*U*_eq_(C). The highest residual electron density peak is located at 0.76 Å from Br1 and the deepest hole is located at 0.69 Å from Br1.

### Crystal data

**Table:**

C_23_H_15_BrO	*F*(000) = 784
*M**_r_* = 387.25	*D*_x_ = 1.544 Mg m^−^^3^
Monoclinic, *P*2_1_/*c*	Mo *K*α radiation, λ = 0.71073 Å
Hall symbol: -P 2ybc	Cell parameters from 4866 reflections
*a* = 5.3792 (1) Å	θ = 2.1–30.0°
*b* = 19.1030 (4) Å	µ = 2.47 mm^−^^1^
*c* = 16.3005 (4) Å	*T* = 100 K
β = 95.944 (1)°	Plate, yellow
*V* = 1666.02 (6) Å^3^	0.57 × 0.27 × 0.15 mm
*Z* = 4	

### Data collection

**Table:**

Bruker SMART APEXII CCD area-detector diffractometer	4866 independent reflections
Radiation source: fine-focus sealed tube	3803 reflections with *I* > 2σ(*I*)
graphite	*R*_int_ = 0.036
Detector resolution: 8.33 pixels mm^-1^	θ_max_ = 30.0°, θ_min_ = 2.1°
ω scans	*h* = −7→7
Absorption correction: multi-scan (*SADABS*; Bruker, 2005)	*k* = −26→26
*T*_min_ = 0.331, *T*_max_ = 0.714	*l* = −22→22
29994 measured reflections	

### Refinement

**Table:**

Refinement on *F*^2^	Primary atom site location: structure-invariant direct methods
Least-squares matrix: full	Secondary atom site location: difference Fourier map
*R*[*F*^2^ > 2σ(*F*^2^)] = 0.032	Hydrogen site location: inferred from neighbouring sites
*wR*(*F*^2^) = 0.076	H-atom parameters constrained
*S* = 1.02	*w* = 1/[σ^2^(*F*_o_^2^) + (0.0322*P*)^2^ + 1.1607*P*] where *P* = (*F*_o_^2^ + 2*F*_c_^2^)/3
4866 reflections	(Δ/σ)_max_ = 0.001
226 parameters	Δρ_max_ = 0.53 e Å^−^^3^
0 restraints	Δρ_min_ = −0.54 e Å^−^^3^

### Special details

**Table:**

Experimental. The low-temperature data was collected with the Oxford Cyrosystem Cobra low-temperature attachment.
Geometry. All e.s.d.'s (except the e.s.d. in the dihedral angle between two l.s. planes) are estimated using the full covariance matrix. The cell e.s.d.'s are taken into account individually in the estimation of e.s.d.'s in distances, angles and torsion angles; correlations between e.s.d.'s in cell parameters are only used when they are defined by crystal symmetry. An approximate (isotropic) treatment of cell e.s.d.'s is used for estimating e.s.d.'s involving l.s. planes.
Refinement. Refinement of *F*^2^ against ALL reflections. The weighted *R*-factor *wR* and goodness of fit *S* are based on *F*^2^, conventional *R*-factors *R* are based on *F*, with *F* set to zero for negative *F*^2^. The threshold expression of *F*^2^ > σ(*F*^2^) is used only for calculating *R*-factors(gt) *etc*. and is not relevant to the choice of reflections for refinement. *R*-factors based on *F*^2^ are statistically about twice as large as those based on *F*, and *R*- factors based on ALL data will be even larger.

### Fractional atomic coordinates and isotropic or equivalent isotropic displacement parameters (Å^2^)

**Table:**

	*x*	*y*	*z*	*U*_iso_*/*U*_eq_	
Br1	0.32584 (4)	0.344013 (10)	0.411244 (11)	0.02530 (7)	
O1	0.9073 (2)	0.51056 (7)	0.74450 (8)	0.0213 (3)	
C1	0.7274 (4)	0.41439 (9)	0.62533 (11)	0.0201 (4)	
H1A	0.8800	0.4043	0.6552	0.024*	
C2	0.6526 (4)	0.37631 (10)	0.55484 (12)	0.0219 (4)	
H2A	0.7530	0.3408	0.5374	0.026*	
C3	0.4261 (4)	0.39203 (9)	0.51095 (10)	0.0180 (4)	
C4	0.2711 (4)	0.44314 (10)	0.53719 (11)	0.0206 (4)	
H4A	0.1176	0.4523	0.5075	0.025*	
C5	0.3464 (3)	0.48075 (10)	0.60836 (10)	0.0184 (4)	
H5A	0.2419	0.5149	0.6267	0.022*	
C6	0.5781 (3)	0.46766 (9)	0.65244 (10)	0.0152 (3)	
C7	0.6813 (3)	0.51052 (9)	0.72478 (10)	0.0159 (3)	
C8	0.5083 (3)	0.55087 (9)	0.77158 (10)	0.0159 (3)	
H8A	0.3374	0.5499	0.7555	0.019*	
C9	0.5978 (3)	0.58858 (9)	0.83690 (10)	0.0161 (3)	
H9A	0.7707	0.5920	0.8471	0.019*	
C10	0.4492 (3)	0.62533 (9)	0.89440 (10)	0.0158 (3)	
C11	0.5151 (3)	0.61533 (9)	0.98020 (10)	0.0157 (3)	
C12	0.7151 (4)	0.57046 (10)	1.01164 (11)	0.0195 (4)	
H12A	0.8015	0.5452	0.9749	0.023*	
C13	0.7822 (4)	0.56385 (10)	1.09428 (11)	0.0219 (4)	
H13A	0.9149	0.5349	1.1132	0.026*	
C14	0.6506 (4)	0.60082 (10)	1.15145 (11)	0.0220 (4)	
H14A	0.7002	0.5970	1.2076	0.026*	
C15	0.4527 (4)	0.64183 (10)	1.12478 (11)	0.0224 (4)	
H15A	0.3652	0.6648	1.1631	0.027*	
C16	0.3767 (3)	0.65030 (9)	1.03860 (10)	0.0169 (3)	
C17	0.1715 (4)	0.69108 (9)	1.01020 (10)	0.0187 (4)	
H17A	0.0769	0.7118	1.0482	0.022*	
C18	0.1036 (3)	0.70178 (9)	0.92633 (10)	0.0167 (3)	
C19	−0.1040 (4)	0.74534 (9)	0.89840 (11)	0.0196 (4)	
H19A	−0.2054	0.7629	0.9365	0.023*	
C20	−0.1560 (4)	0.76158 (9)	0.81740 (11)	0.0211 (4)	
H20A	−0.2925	0.7898	0.8002	0.025*	
C21	−0.0008 (4)	0.73530 (9)	0.75909 (11)	0.0205 (4)	
H21A	−0.0309	0.7487	0.7041	0.025*	
C22	0.1913 (3)	0.69077 (9)	0.78224 (10)	0.0184 (4)	
H22A	0.2862	0.6730	0.7424	0.022*	
C23	0.2504 (3)	0.67066 (9)	0.86689 (10)	0.0153 (3)	

### Atomic displacement parameters (Å^2^)

**Table:**

	*U*^11^	*U*^22^	*U*^33^	*U*^12^	*U*^13^	*U*^23^
Br1	0.03907 (13)	0.02093 (10)	0.01575 (9)	−0.00663 (8)	0.00217 (7)	−0.00386 (7)
O1	0.0137 (7)	0.0297 (7)	0.0203 (6)	0.0009 (5)	0.0009 (5)	−0.0031 (5)
C1	0.0181 (10)	0.0199 (9)	0.0219 (9)	0.0037 (7)	0.0000 (7)	−0.0002 (7)
C2	0.0238 (10)	0.0180 (9)	0.0239 (9)	0.0060 (7)	0.0029 (7)	−0.0027 (7)
C3	0.0238 (10)	0.0159 (8)	0.0146 (8)	−0.0052 (7)	0.0032 (6)	−0.0018 (6)
C4	0.0170 (10)	0.0258 (9)	0.0182 (8)	−0.0003 (7)	−0.0010 (7)	−0.0016 (7)
C5	0.0158 (9)	0.0228 (9)	0.0168 (8)	0.0022 (7)	0.0020 (6)	−0.0031 (7)
C6	0.0155 (9)	0.0169 (8)	0.0134 (7)	−0.0013 (6)	0.0024 (6)	0.0007 (6)
C7	0.0156 (9)	0.0176 (8)	0.0148 (7)	−0.0011 (7)	0.0027 (6)	0.0005 (6)
C8	0.0119 (9)	0.0206 (8)	0.0155 (7)	−0.0010 (7)	0.0022 (6)	−0.0008 (6)
C9	0.0133 (9)	0.0179 (8)	0.0175 (8)	−0.0016 (7)	0.0032 (6)	0.0007 (6)
C10	0.0157 (9)	0.0163 (8)	0.0155 (8)	−0.0035 (7)	0.0024 (6)	−0.0019 (6)
C11	0.0163 (9)	0.0155 (8)	0.0154 (8)	−0.0034 (7)	0.0018 (6)	−0.0006 (6)
C12	0.0195 (10)	0.0211 (9)	0.0184 (8)	−0.0005 (7)	0.0034 (7)	−0.0008 (7)
C13	0.0209 (10)	0.0240 (9)	0.0202 (8)	−0.0003 (8)	−0.0003 (7)	0.0026 (7)
C14	0.0292 (11)	0.0223 (9)	0.0140 (8)	−0.0036 (8)	−0.0004 (7)	0.0005 (7)
C15	0.0307 (11)	0.0227 (9)	0.0141 (8)	−0.0017 (8)	0.0042 (7)	−0.0017 (7)
C16	0.0210 (9)	0.0144 (8)	0.0156 (8)	−0.0040 (7)	0.0036 (6)	−0.0015 (6)
C17	0.0225 (10)	0.0168 (8)	0.0175 (8)	−0.0003 (7)	0.0054 (7)	−0.0032 (6)
C18	0.0182 (9)	0.0142 (8)	0.0178 (8)	−0.0032 (7)	0.0021 (6)	−0.0015 (6)
C19	0.0193 (10)	0.0156 (8)	0.0244 (9)	−0.0002 (7)	0.0050 (7)	−0.0024 (7)
C20	0.0200 (10)	0.0160 (8)	0.0266 (9)	−0.0010 (7)	−0.0018 (7)	0.0006 (7)
C21	0.0216 (10)	0.0209 (9)	0.0182 (8)	−0.0033 (7)	−0.0016 (7)	0.0014 (7)
C22	0.0192 (10)	0.0191 (9)	0.0168 (8)	−0.0029 (7)	0.0020 (6)	−0.0020 (7)
C23	0.0155 (9)	0.0154 (8)	0.0150 (7)	−0.0048 (6)	0.0013 (6)	−0.0018 (6)

### Geometric parameters (Å, °)

**Table:**

Br1—C3	1.8954 (17)	C12—C13	1.364 (2)
O1—C7	1.225 (2)	C12—H12A	0.93
C1—C2	1.384 (3)	C13—C14	1.416 (3)
C1—C6	1.396 (2)	C13—H13A	0.93
C1—H1A	0.93	C14—C15	1.356 (3)
C2—C3	1.380 (3)	C14—H14A	0.93
C2—H2A	0.93	C15—C16	1.431 (2)
C3—C4	1.380 (3)	C15—H15A	0.93
C4—C5	1.389 (2)	C16—C17	1.391 (3)
C4—H4A	0.93	C17—C18	1.393 (2)
C5—C6	1.395 (2)	C17—H17A	0.93
C5—H5A	0.93	C18—C19	1.429 (3)
C6—C7	1.495 (2)	C18—C23	1.440 (2)
C7—C8	1.480 (2)	C19—C20	1.357 (3)
C8—C9	1.333 (2)	C19—H19A	0.93
C8—H8A	0.93	C20—C21	1.420 (3)
C9—C10	1.472 (2)	C20—H20A	0.93
C9—H9A	0.93	C21—C22	1.361 (3)
C10—C23	1.413 (3)	C21—H21A	0.93
C10—C11	1.420 (2)	C22—C23	1.436 (2)
C11—C12	1.428 (3)	C22—H22A	0.93
C11—C16	1.433 (2)		
			
C2—C1—C6	121.23 (17)	C11—C12—H12A	119.3
C2—C1—H1A	119.4	C12—C13—C14	120.32 (18)
C6—C1—H1A	119.4	C12—C13—H13A	119.8
C3—C2—C1	118.75 (17)	C14—C13—H13A	119.8
C3—C2—H2A	120.6	C15—C14—C13	120.41 (17)
C1—C2—H2A	120.6	C15—C14—H14A	119.8
C4—C3—C2	121.49 (16)	C13—C14—H14A	119.8
C4—C3—Br1	118.73 (14)	C14—C15—C16	121.00 (17)
C2—C3—Br1	119.78 (14)	C14—C15—H15A	119.5
C3—C4—C5	119.44 (17)	C16—C15—H15A	119.5
C3—C4—H4A	120.3	C17—C16—C15	121.78 (16)
C5—C4—H4A	120.3	C17—C16—C11	119.28 (16)
C4—C5—C6	120.34 (17)	C15—C16—C11	118.94 (17)
C4—C5—H5A	119.8	C16—C17—C18	121.81 (16)
C6—C5—H5A	119.8	C16—C17—H17A	119.1
C5—C6—C1	118.69 (16)	C18—C17—H17A	119.1
C5—C6—C7	123.18 (16)	C17—C18—C19	120.98 (16)
C1—C6—C7	118.04 (16)	C17—C18—C23	119.57 (16)
O1—C7—C8	121.62 (16)	C19—C18—C23	119.41 (15)
O1—C7—C6	119.02 (15)	C20—C19—C18	121.24 (17)
C8—C7—C6	119.35 (15)	C20—C19—H19A	119.4
C9—C8—C7	119.93 (16)	C18—C19—H19A	119.4
C9—C8—H8A	120.0	C19—C20—C21	119.62 (18)
C7—C8—H8A	120.0	C19—C20—H20A	120.2
C8—C9—C10	126.25 (17)	C21—C20—H20A	120.2
C8—C9—H9A	116.9	C22—C21—C20	121.16 (16)
C10—C9—H9A	116.9	C22—C21—H21A	119.4
C23—C10—C11	119.94 (15)	C20—C21—H21A	119.4
C23—C10—C9	122.24 (15)	C21—C22—C23	121.31 (17)
C11—C10—C9	117.80 (16)	C21—C22—H22A	119.3
C10—C11—C12	122.43 (16)	C23—C22—H22A	119.3
C10—C11—C16	119.84 (16)	C10—C23—C22	123.65 (16)
C12—C11—C16	117.72 (15)	C10—C23—C18	119.29 (15)
C13—C12—C11	121.49 (17)	C22—C23—C18	116.99 (16)
C13—C12—H12A	119.3		
			
C6—C1—C2—C3	0.3 (3)	C13—C14—C15—C16	−1.8 (3)
C1—C2—C3—C4	−1.9 (3)	C14—C15—C16—C17	178.78 (18)
C1—C2—C3—Br1	176.90 (14)	C14—C15—C16—C11	−0.8 (3)
C2—C3—C4—C5	1.4 (3)	C10—C11—C16—C17	3.0 (3)
Br1—C3—C4—C5	−177.43 (14)	C12—C11—C16—C17	−176.19 (16)
C3—C4—C5—C6	0.8 (3)	C10—C11—C16—C15	−177.41 (16)
C4—C5—C6—C1	−2.4 (3)	C12—C11—C16—C15	3.4 (2)
C4—C5—C6—C7	174.05 (17)	C15—C16—C17—C18	177.22 (17)
C2—C1—C6—C5	1.9 (3)	C11—C16—C17—C18	−3.2 (3)
C2—C1—C6—C7	−174.75 (17)	C16—C17—C18—C19	−178.36 (17)
C5—C6—C7—O1	−158.07 (17)	C16—C17—C18—C23	−0.7 (3)
C1—C6—C7—O1	18.4 (2)	C17—C18—C19—C20	173.37 (17)
C5—C6—C7—C8	22.9 (2)	C23—C18—C19—C20	−4.3 (3)
C1—C6—C7—C8	−160.59 (16)	C18—C19—C20—C21	−0.4 (3)
O1—C7—C8—C9	0.1 (3)	C19—C20—C21—C22	3.8 (3)
C6—C7—C8—C9	179.10 (16)	C20—C21—C22—C23	−2.3 (3)
C7—C8—C9—C10	−173.26 (16)	C11—C10—C23—C22	171.79 (16)
C8—C9—C10—C23	−50.2 (3)	C9—C10—C23—C22	−6.7 (3)
C8—C9—C10—C11	131.30 (19)	C11—C10—C23—C18	−5.1 (3)
C23—C10—C11—C12	−179.68 (17)	C9—C10—C23—C18	176.46 (16)
C9—C10—C11—C12	−1.1 (3)	C21—C22—C23—C10	−179.25 (18)
C23—C10—C11—C16	1.1 (3)	C21—C22—C23—C18	−2.3 (3)
C9—C10—C11—C16	179.69 (16)	C17—C18—C23—C10	4.9 (3)
C10—C11—C12—C13	177.21 (17)	C19—C18—C23—C10	−177.42 (16)
C16—C11—C12—C13	−3.6 (3)	C17—C18—C23—C22	−172.15 (16)
C11—C12—C13—C14	1.1 (3)	C19—C18—C23—C22	5.5 (2)
C12—C13—C14—C15	1.6 (3)		

### Hydrogen-bond geometry (Å, °)

**Table:**

*D*—H···*A*	*D*—H	H···*A*	*D*···*A*	*D*—H···*A*
C8—H8A···O1^i^	0.93	2.42	3.308 (2)	159
C13—H13A···O1^ii^	0.93	2.57	3.288 (2)	135
C21—H21A···Br1^iii^	0.93	2.93	3.4722 (19)	119
C9—H9A···Cg1^iv^	0.93	2.83	3.4479 (18)	125

Symmetry codes: (i) *x*−1, *y*, *z*; (ii) −*x*+2, −*y*+1, −*z*+2; (iii) −*x*, −*y*+1, −*z*+1; (iv) *x*+1, *y*, *z*.
